# Land Classification and Change Intensity Analysis in a Coastal Watershed of Southeast China

**DOI:** 10.3390/s140711640

**Published:** 2014-07-01

**Authors:** Pei Zhou, Jinliang Huang, Pontius Robert Gilmore, Huasheng Hong

**Affiliations:** 1 Coastal and Ocean Management Institute, Xiamen University, Xiamen 361005, China; E-Mails: PeiZhou.0419@gmail.com (P.Z.); hshong@xmu.edu.cn (H.H.); 2 Fujian Provincial Key Laboratory of Coastal Ecology and Environmental Studies, Xiamen University, Xiamen 361005, China; 3 Graduate School of Geography, Clark University, Worcester, MA 01610, USA; E-Mail: rpontius@clarku.edu

**Keywords:** land-use and land-cover, stratified classification, intensity analysis, coastal watershed

## Abstract

The aim of this study is to improve the understanding of land changes in the Jiulong River watershed, a coastal watershed of Southeast China. We developed a stratified classification methodology for land mapping, which combines linear stretching, an Iterative Self-Organizing Data Analysis (ISODATA) clustering algorithm, and spatial reclassification. The stratified classification for 2002 generated less overall error than an unstratified classification. The stratified classifications were then used to examine temporal differences at 1986, 1996, 2002, 2007 and 2010. Intensity Analysis was applied to analyze land changes at three levels: time interval, category, and transition. Results showed that land use transformation has been accelerating. Woodland's gains and losses were dormant while the gains and losses of Agriculture, Orchard, Built-up and Bare land were active during all time intervals. Water's losses were active and stationary. The transitions from Agriculture, Orchard, and Water to Built-up were systematically targeting and stationary, while the transition from Woodland to Built-up was systematically avoiding and stationary.

## 1. Introduction

Coastal watersheds have become a particular concern due to escalating environmental pressures and their important roles in coastal ecosystem services such as fresh water provision and nutrient cycling supporting services [1,2]. The pollutants in coastal watersheds transported from rivers are blamed for contamination of downstream estuarine and coastal waters [3–5]. Watershed land use influences water quality through nonpoint sources, which are major contributors of pollution to the catchment-coast continuum under the context of human-accelerated landscape transformations [2,3,6,7]. As human activities determine land use and land cover (LULC) pattern, accurate mapping of LULC and thereby understanding how LULC might evolve is crucial for watershed assessment and land-based pollution management [8–10].

Satellite remote sensing has the potential to provide geospatial information describing changes in LULC [10–12]. A number of techniques on LULC classification and change detection have been reviewed [13–15]. LULC classification approaches mainly include supervised classification and unsupervised classification [16]. Classification performance depends on many factors [17,18]. For example, supervised classification relies on previous knowledge and accurately located training samples. Classifying satellite images and extracting meaningful information in an efficient way without compromising the accuracy has remained a challenge even though a variety of remotely sensed sources with various spatial, temporal and spectral scales are available [10,19].

Spectral confusion is a challenge in land cover mapping, especially when using a spectral-based classification method. Yang and Lo [20] identified spectral similarity between urban features and various vegetation types. The confused clusters were further split and recoded. Liu and Yang [21] tackled the spectral confusion problem by employing a stratified classification strategy. They partitioned the entire landscape into rural and urban subsets using the road network density. Then they processed those subsets independently to minimize the spectral confusion between some urban features and agricultural land covers. This stratified classification was used in an urban area and used the road buffer as a mask to clip the entire landscape into two subsets. The spectral feature itself should be taken into account when making the mask to partition satellite images.

Post classification comparison is a commonly used method for change detection [22]. An obvious first step to quantifying the change among land categories between two points in time is to compute the transition matrix. Pontius *et al.* [23] created a method to examine the transitions relative to the sizes of the categories available for the transitions. Alo and Pontius [24] showed how to use the method to test whether the systematic transitions in one region are consistent with the systematic transitions in a different region for cases where the two regions have different proportions of the stocks of land categories. More recently, Aldwaik and Pontius [25] developed the method further into an approach called Intensity Analysis, which examines changes among categories at three levels: time interval, category and transition. Intensity Analysis quantifies at each level the deviation between observed change intensity and hypothesized uniform change intensity. Many researchers [26–29] have used the concepts introduced by Pontius *et al.* [23]. Huang *et al.* adopted Intensity Analysis to link patterns with process of LULC change in the Jiulong River Watershed (JRW) [30].

The JRW is a subtropical watershed located in the eastern coastal area of China that has experienced drastic LULC changes during the past 20 years and that plays an important role in the region's economic and ecological health. However, studies that use accurate and detailed mapping of LULC and its changes over time in this coastal watershed are needed. The objectives of our study are: (i) to develop a stratified classification methodology to create a time series of LULC maps in the JRW, and (ii) to analyze the spatiotemporal dynamics of LULC in the JRW.

## 2. Study Area

**Figure 1.**
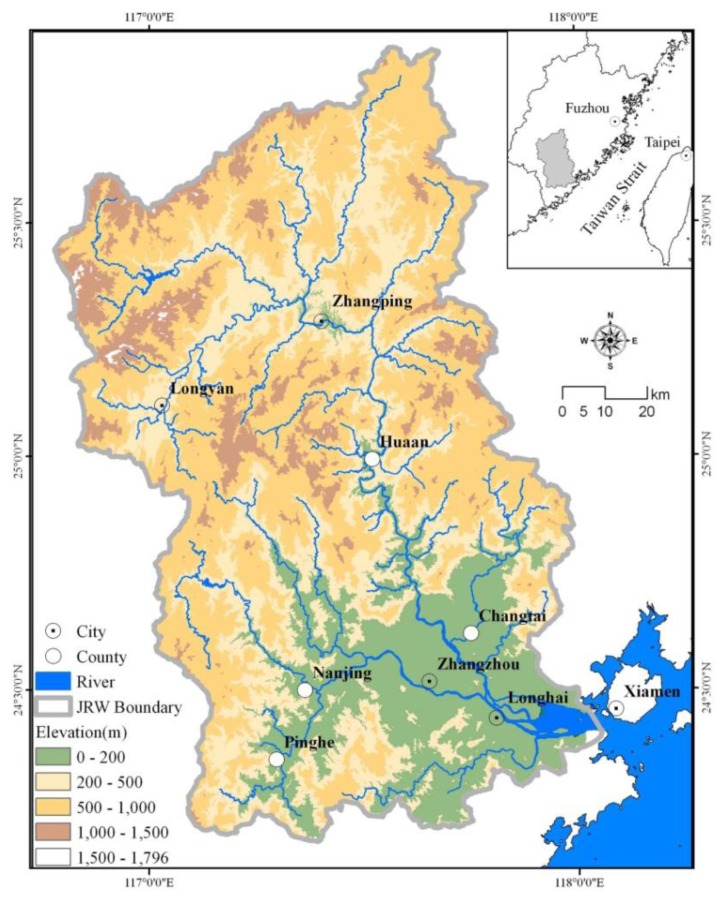
Location of the Jiulong River Watershed.

The JRW covers about 14,700 km^2^ in the eastern coastal area of China and consists mainly of eight counties/districts: Zhangzhou, Xinlou, Zhangping, Hua'an, Changtai, Pinghe, Longhai and Nangjing (Figure 1). The watershed's gross domestic product accounts for a quarter of Fujian Province's economic output. Approximately ten million residents from Xiamen, Zhangzhou and Longyan use the Jiulong River as their water source for residential, industrial and agricultural activities.

The watershed includes the North and West Rivers, which meet in Zhangzhou, and produce an annual flow of twelve billion cubic meters into the Jiulong River estuary and the Xiamen-Kinmen coast. The upstream region is mountainous and 68% of the watershed has a topographic slope in excess of 18% [31]. Fujian Province's largest plain is the Zhangzhou plain, which is located at the downstream end of the Jiulong River. The plain is intensively agricultural with orchards of banana, longan, litchi, pomelo, citrus and flowers. The Zhangzhou municipality, including the Zhangzhou district and its neighboring counties (Longhai, Changtai, Nanjing, and Pinghe), constitutes one of China's most developed regions in terms of agricultural production due to its subtropical monsoon climate and agricultural policies, which are influenced by the closeness to Taiwan.

## 3. Data and Methodology

The rare availability of cloud free imagery influenced the selection of the dates of the images. Inconsistency in acquisition date within the same year can cause inconsistency in the classification for that year. After examining available satellite images, we realized that the problems that clouds would cause would be much larger than problems that inconsistent date acquisition would cause. Therefore, for each year, two images from different dates were chosen to obtain a complete and cloud free coverage of the study area (Table 1). The predominantly cloud-free Landsat images covering the inland and estuarine parts of the JRW included eight Thematic Mapper (TM) images acquired in 1986, 1996, 2007, 2008, 2009 and 2010 and two Enhanced Thematic Mapper Plus (ETM+) images acquired in 2002. These Landsat images were acquired from the Center for Earth Observation and Digital Earth (CEODE), Chinese Academy of Sciences (http://cs.rsgs.ac.cn/cs_cn/) and the United States Geological Survey (USGS) (http://eros.usgs.gov/). In order to keep the same resolution, the panchromatic band of ETM+ image was not used and all the TM images were re-sampled to a 30 m resolution. The final LULC map for each year is a mosaic of classified maps of the inland and estuarine parts. Ancillary data included the high-resolution image from Google Earth and GIS data of roads and villages. These geospatial data were used mainly as reference data for image classification and accuracy assessment.

**Figure 2.**
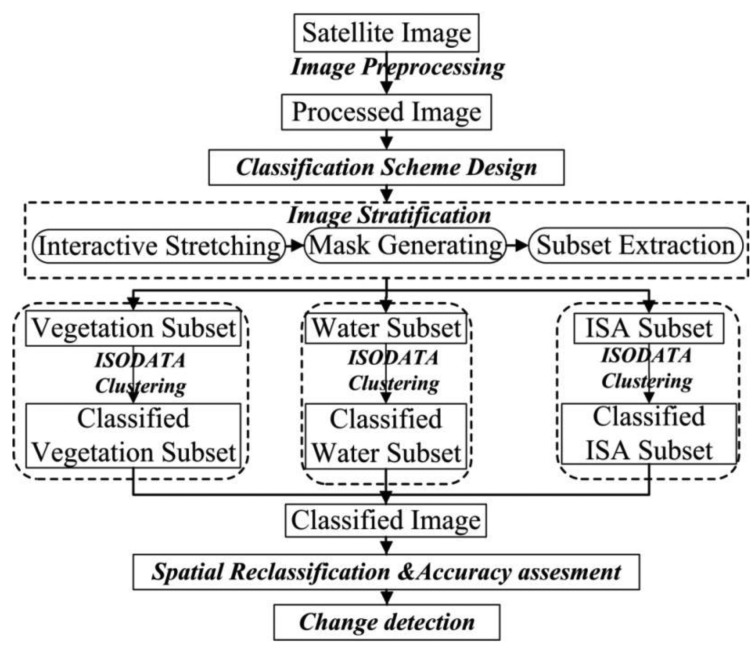
Seven steps of methodology.

Our methodology proceeded in seven steps (Figure 2): (i) image preprocessing; (ii) LULC classification scheme design; (iii) image stratification; (iv) ISODATA clustering; (v) spatial reclassification; (vi) accuracy assessment and (vii) change detection. LULC classes in the study area were summarized into six categories: Woodland, Agriculture, Orchard, Built-up, Bare land and Water (Table 2). In addition, we classified two ETM+ images in 2002 using the conventional unsupervised classification procedure without the image stratification procedure so we could compare it to the output of the stratified procedure, to measure the effect of the stratification.

### 3.1. Image Preprocessing

In this study, the geo-referencing approach was image-to-image registration. First, the 2002 ETM+ image was registered to topographic maps using distinctive features, such as road intersections and stream confluences that are clearly visible. The ETM+ image was georeferenced to the Krasovsky 1940 map projection, Beijing 1954 coordinate system, Central Meridian 117 N. Then, this image was used as the reference to rectify other images. For the four cases, a first-order polynomial nearest neighbor algorithm with 32 ground control points re-sampled the images so that the root mean square errors were less than half a pixel. All the images were re-sampled to a 30 m resolution before the following steps.

### 3.2. LULC Classification Scheme

The Technology Regulation of Land Use Survey (1984), regulated by the Ministry of Land and Resources of the People's Republic of China, recommends that LULC be classified into Agriculture, Orchard, Woodland, Grassland, Residential area, Transportation, Water and Bare land. We created a modified version of six categories of LULC based on image spatial resolution and field surveys (Table 2).

### 3.3. Image Stratification

Spectral features based image stratification was developed in this study to suppress the spectral confusion. Electromagnetic radiation reflection from landmarks is the basis of remotely sensed images and the lightness of the pixels depends on the landmarks, by which specific landmarks can be identified [32–34]. It is well known that the red band locates in the main absorption band of chlorophyll, and contains most information on vegetation. Water body is distinguishable from background in the short-wave infrared band [32]. Therefore, the red band and short-wave infrared band are used in this study to extract the vegetation and water layer through the digital number (DN) value thresholds. The image stratification performed in this study consists of the following steps:

(1) Linear stretching. Interactive stretching allows users to interactively control the contrast of the displayed image. Here, we used the linear stretching and adjusted the DN value of the red band to control the contrast of vegetation and other land cover. The optimal DN value here separated distinctly vegetation from other land cover types and was defined as the vegetation threshold in this work. Similarly, by adjusting the DN value of the short-wave infrared band and controlling the contrast of water and other land cover in the short-wave infrared band, we obtained a threshold for water.
(2) Mask generating. If the DN value in the red band of a pixel was smaller than the vegetation threshold we obtained, then this pixel was recognized as part of the mask for vegetation. All the pixels under above-mentioned condition composed the mask for vegetation subset. Similarly, if the DN value in the short-wave infrared band of a pixel was smaller than the water threshold we obtained, then this pixel was recognized as part of the mask for water subset. All the pixels under above-mentioned condition composed the mask for water subset.
(3) Subset extracting. The vegetation subset was partitioned from the processed image using the vegetation mask. Here we got a vegetation subset and remaining image. Then by clipping the remaining image using the water mask, we got a water subset and another subset, which consisted mainly of urban area. We named the third subset impervious surface areas (ISA).

### 3.4. ISODATA Clustering

Then the ISODATA classifier was used to identify spectral clusters. The number of classes is critical for ISODATA classifier to capture the land surface variability from images. Yang and Lo [20] empirically tried various numbers of classes to determine the optimum number. If the resultant clusters are better interpreted in relation to the classification scheme, then the corresponding number is recognized as the optimum. According to the above-mentioned method, water, ISA and vegetation subsets were grouped into 40, 60 and 150 spectral clusters. The number of Maximum Iterations was specified as 60, and the Convergence Threshold was specified as 0.990.

Each spectral cluster in all three subsets was assigned into one of the six LULC classes using visual interpretation of the subset images, and the ancillary data from Google Earth and GIS data were also used. A single LULC image for each year was generated by merging the three images. After generating the classified inland and estuarine maps, these two were mosaicked and clipped according to our study area boundary.

### 3.5. Spatial Reclassification

Manual on-screen editing was applied to further correct the classified image. The classification used ancillary data, including finer resolution satellite imagery data from Google Earth, the original images, the authors' knowledge of the study area, and GIS data of roads and villages. Finally the LULC map was clipped by the mask of our study area boundary.

### 3.6. Accuracy Assessment

A stratified random sampling design was adopted in the accuracy assessment, where the sample size in each stratum was proportional to the size of the stratum, and each stratum is a category in the classified map. For each LULC map, a total of 256 pixels were selected. We checked each pixel by visual inspection of the Landsat images and fine resolution images from Google Earth to construct a confusion matrix.

We summarized the confusion matrices by computing quantity disagreement and allocation disagreement [35]. Quantity disagreement is the amount of difference between the reference categories and the classified categories that is due to the less than perfect match in the proportions of the categories. Allocation disagreement is defined as the amount of difference between the reference categories and the classified categories that is due to the less than maximum match in the spatial allocation of the categories, given the proportions of the categories in the reference and comparison maps. The total disagreement is the sum of quantity disagreement and allocation disagreement.

In terms of each category, agreement for a particular category is where both the reference information and the classified map indicate the particular category. Omission disagreement for a particular category is where the reference information indicates the particular category, but the classified map shows a different category. Commission disagreement for a particular category is where the classified map shows the particular category, but the reference information indicates a different category. If the commission disagreement is greater than omission disagreement for a particular category, then the map overestimates the quantity of that category. If the omission disagreement is greater than commission disagreement for a particular category, then the map underestimates the quantity of that category [36].

### 3.7. Change Detection

Change detection was carried out by post-classification comparison, and creation of contingency tables for the time intervals 1986–1996, 1996–2002, 2002–2007 and 2007–2010. We performed three levels of Intensity Analysis: time interval, category, and transition [25]. The time interval level examines how the size and rate of change varies across time intervals. For any particular time interval, the category level examines how the size and intensity of gross losses and gross gains in each category vary across categories. For any particular category, the transition level examines how the size and intensity of the category's transitions vary across the other categories that are available for that transition. At each level, Intensity Analysis compares the observed changes to hypothetical uniform change, as a uniform line indicates.

At each level, the method tests for the stationarity of patterns across time intervals. *Stationary* means that the pattern of change in one time interval is the same as the pattern of change in a different time interval. For the category level of the gains, stationary means that the intensity of a category's gain is either greater than the uniform line for all intervals of less than the uniform line for all intervals. Similarly, if the intensity of a category's loss is either greater than the uniform line for all intervals or less than the uniform line for all intervals, then that category is stationary in terms of losses. For the transition analysis, if the gain of category *n* either targets category *m* for all time intervals or avoids category *m* for all time intervals, then the transition from *m* to *n* is stationary, given the gain of *n*. Similarly, if the loss of category *m* is either avoided by category *n* for all time intervals or targeted by category *n* for all time intervals, then we define the transition from *m* to *n* as stationary, given the loss of *m*.

According to the definition of Alo and Pontius [24], the transition from category *m* to category *n* is a systematically targeting transition when the gain of *n* targets *m* while *n* targets the loss of *m*. The transition from category *m* to category *n* is a systematically avoiding transition when the gain of *n* targets *m* while *n* targets the loss of *m*.

## 4. Results

### 4.1. Performance of Stratified Classification Methodology

**Figure 3.**
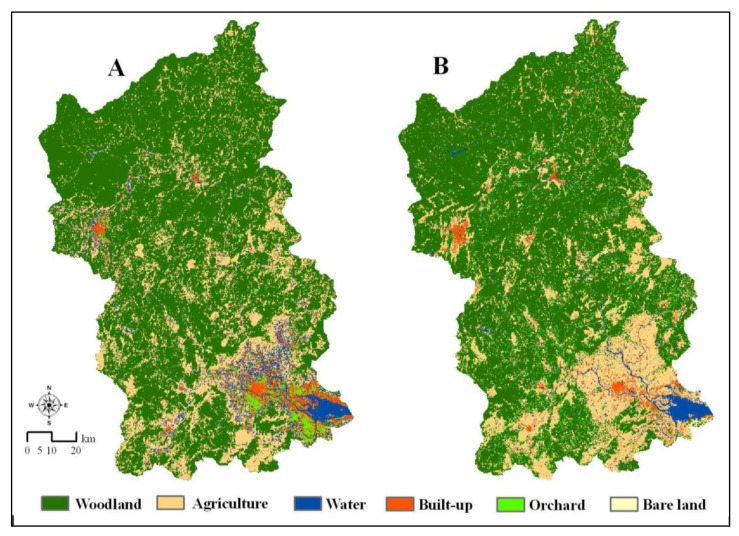
LULC maps of 2002 for the Jiulong River Watershed classified by (**A**) stratified and (**B**) unstratified classification.

Figure 3 shows LULC classification maps in 2002 derived from two methods. Water and Built-up were distinctly classified when using the stratified classification, by which water and impervious surface area were stratified out of other LULC categories.

**Figure 4.**
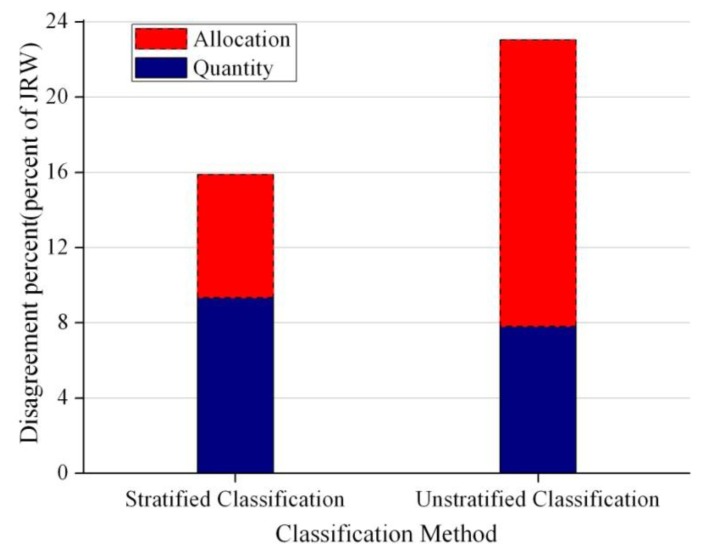
Components of disagreement between LULC maps of 2002 and the reference data.

Figure 4 summarizes the disagreement between the LULC map in 2002 and the reference information, for each of the two methods to produce the maps. Stratified classification produced higher quantity disagreement percent but distinctly smaller total disagreement.

**Figure 5.**
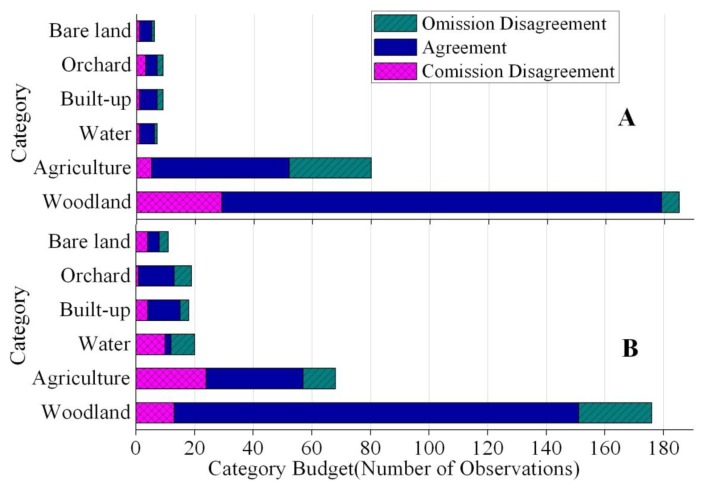
Category level analysis of agreement, omission disagreement and commission disagreement for LULC maps of 2002 classified by (**A**) stratified and (**B**) unstratified classification.

Figure 5 summarizes the agreement, omission disagreement and commission disagreement by category for each of the stratified and unstratified 2002 LULC maps. Vertical axis shows the categories while the horizontal axis shows the number of validation observations. The sum of omission and commission disagreement for the stratified classification is smaller than the sum for the unstratified classification for each category. Figure 5A shows that the commission disagreement is greater than omission disagreement for Woodland and Orchard, thus those two LULC types were overestimated by the stratified classification method. In contrast, Agriculture and Built-up were underestimated. Figure 5B shows that all LULC types except for Woodland and Built-up were overestimated when using the unstratified classification.

This method was further applied to classify TM imagery in 1986, 1996, 2007 and 2010. The overall agreement for 1986, 1996, 2007 and 2010 are 82%, 89%, 83% and 83%.

### 4.2. Spatiotemporal Dynamics of LULC Changes Using Intensity Analysis at Three Levels

**Figure 6.**
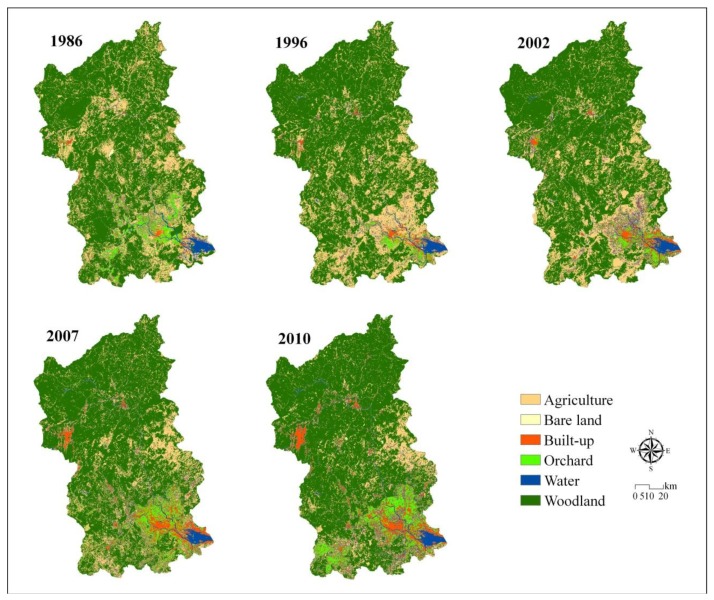
LULC maps of the JRW in 1986, 1996, 2002, 2007 and 2010.

Figure 6 shows maps from the stratified method for five time points. Built-up increased while Agriculture decreased during all time intervals. Orchard increased since 1996. Woodland increased except during 2002–2010.

**Figure 7.**
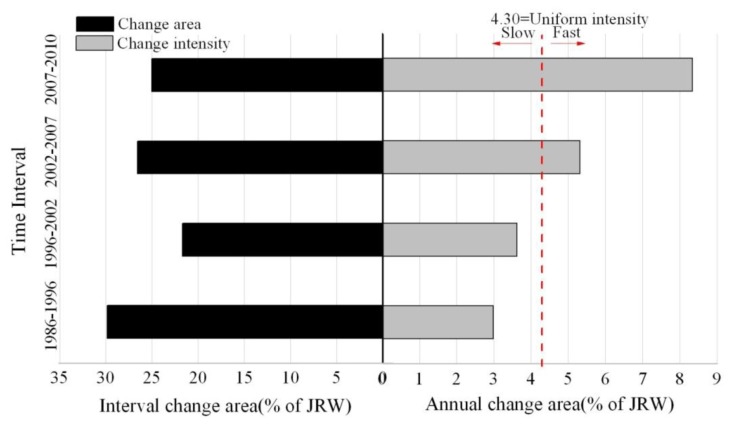
Interval level intensity analysis across four time intervals.

The interval level intensity analysis produced Figure 7. Each bar that extends to the left from the middle axis is the change area. Each bar that extends to the right from the middle axis is the observed change intensity. In terms of the right side of middle axis, if an interval's bar ends before the uniform line, then the change is relatively slow for that interval; if an interval's bar extends beyond the uniform line, then the change is relatively fast for that interval. Figure 7 shows that overall land change has been accelerating across the four time intervals. Annual change is fastest during 2007–2010 though interval change area was the smallest.

**Figure 8.**
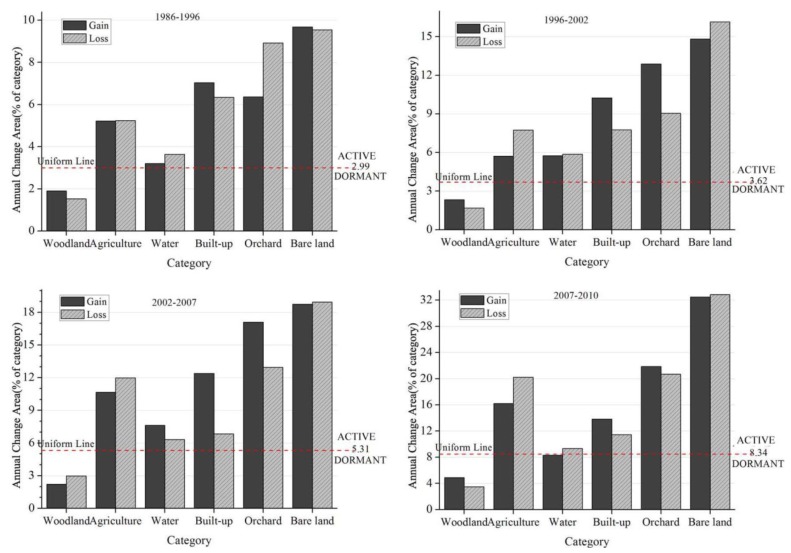
Category level intensity analysis, given the observed change during four time intervals.

The category level intensity analysis for each time interval produced Figure 8. Each category has a pair of bars, where one bar shows gross gain and the other shows gross loss. The vertical axis shows the intensity of annual change during the time interval as a percent of the category. A horizontal line shows a uniform intensity of annual change for the entire study area. If a bar ends below the uniform line, then the change is relatively dormant for that category. If a bar extends above the uniform line, then the change is relatively active for that category.

Woodland's gains and losses were dormant while the gains and losses of Agriculture, Orchard, Built-up and Bare land were active for all time intervals. This indicated that Woodland experienced less intensively gains and losses than if the overall change were to have been distributed uniformly across the landscape. Similarly, Agriculture, Orchard, Built-up and Bare land experienced more intensively gains and losses than if the overall change were to have been distributed uniformly across the landscape. These results are consistent for all four time intervals, meaning that the pattern is stationary at the category level intensity analysis. Water experienced gains more intensively than the landscape in general except for 2007–2010 and experienced losses more intensively than landscape in general across four time intervals. This indicates that Water's losses were stationary while its gains were not.

**Figure 9.**
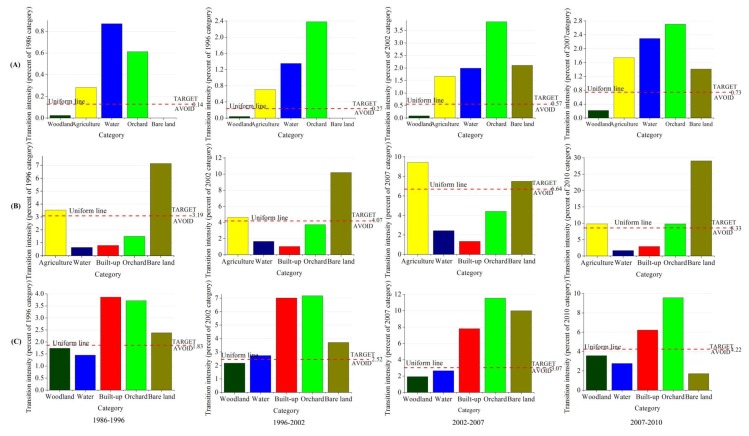

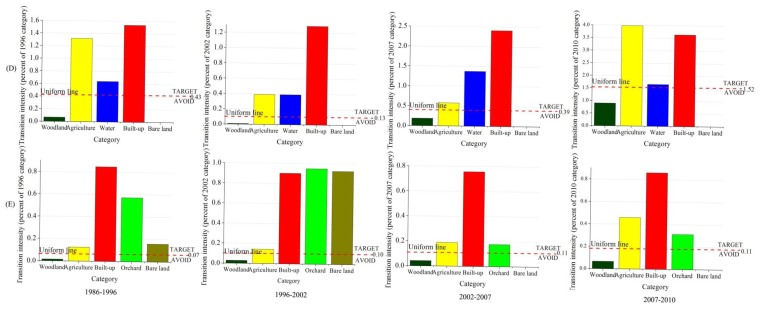
Transition intensities from Woodland, Agriculture, Orchard and Water to Built-up, where figures in row A represent gains to Built-up during the intervals 1986–1996, 1996–2002, 2002–2007 and 2007–2010, respectively, while row B, C, D and E represent losses from Woodland, Agriculture, Orchard and Water during the same intervals respectively.

In terms of transition level intensity analysis, we focused the transitions from Woodland, Agriculture, Orchard and Water to Built-up, as urbanization is a hotspot in the coastal regions throughout the world. Figure 9 shows the results for the transition level analysis in terms of transitions from Woodland, Agriculture, Orchard and Water to Built-up. The four graphs in Figure 9A show the analysis of the gain to Built-up. The horizontal axis shows the losing categories and the vertical axis shows the transition intensity to Built-up. The four lower rows in Figure 9 show the analysis of the loss of Woodland (B), Agriculture (C), Orchard (D) and Water (E). The horizontal axis shows the gaining categories and the vertical axis shows the transition intensity. Figure 9A shows that Built-up's gains target Agriculture, Water and Orchard and avoid Woodland for all time intervals. Therefore the transition from Woodland, Agriculture, Orchard and Water to Built-up is stationary, given the gain of Built-up. Figure 9B shows that Built-up avoids Woodland's loss for all time intervals. The three lower rows of Figure 9 show that Built-up targets the losses of Agriculture, Orchard and Water respectively for all time intervals. The transition from Woodland, Agriculture, Orchard and Water to Built-up is stationary, given the loss of Woodland, Agriculture, Orchard and Water respectively. In Figure 9E, Built-up generally targets Water's loss more intensively than the loss of other categories. This may reflect extensive reclamation in the Jiulong River estuaries in recent years. Intensity Analysis shows that transitions from Agriculture to Built-up, from Orchard to Built-up and from Water to Built-up are systematically targeting transition while the transition from Woodland to Built-up is a systematically avoiding transition.

## 5. Discussion

### 5.1. The Performance of the Stratified Classification

The determinants of LULC classification performance include the classification algorithm, characteristics of the study area, the classification scheme, the pixel spatial resolution, and the quality of the reference data [37]. Stratified classification in this paper extracted subsets based on spectral features and then classified each subset in a same scheme. This is different with hierarchical classification, which classified images in hierarchical way [38,39]. Other studies have also used stratified classification and spatial reclassification procedures to suppress the confusion in spectral signals [21,40]. Liu and Yang [21] stratified the entire landscape into rural and urban subsets and then classified each subset independently by using multiple endmember spectral mixture analysis. The completeness and temporal accuracy of the road network data are critical for the success of landscape stratification in Liu and Yang's study. Unfortunately, we were not able to obtain accurate road network data for the same year as the satellite images. Instead, we took the spectral features of vegetation in the red band and water in the short-wave infrared band to make two masks. The image was then partitioned into three subsets. Therefore, our image stratification doesn't need ancillary data to clip the image. We adopted ISODAT clustering to process each subset, because we were not able to gather enough previous information about our study area.

The stratified classification produced lower overall error than the unstratified classification. However, the stratified classification produced higher quantity disagreement (Figure 4). This was mainly caused by the overestimation of Woodland and underestimation of Agriculture (Figure 5). Specifically, the largest confusion in the stratified map is that the map shows Woodland where the reference information shows Agriculture. Most of the error in the stratified map is due to this single type of confusion.

### 5.2. Spatialtemporal Dynamics of LULC Change in the JRW

Intensity Analysis showed that land transformation has been accelerating across the four time intervals, which is consistent with accelerating economic development in this coastal watershed of Southeast China. This finding is similar to our prior study [41].

Woodland experienced dormant gains and losses, which exemplifies the large dormant category phenomenon [42]. Agriculture, Orchard, Built-up and Bare land experienced active gains and losses. These patterns are stationary at the category level intensity analysis. Our prior study had similar observation that Agriculture and Built were active whereas Natural was a large dormant category [41].

The systematically targeting transitions from Agriculture to Built-up, Orchard to Built-up and Water to Built-up might be attributable to the spatial proximity of those losing categories to urbanization in recent years in the JRW. Towns tend to be in the flat areas, and agricultural activities have historically also been located in these areas. Orchard is traditionally planted in the Zhangzhou plain within downstream of the JRW. Therefore, as the Built-up expands spatially, it is likely to take over Agriculture and Orchard. This explains why most gain of Built land surrounding the cities such as Zhangzhou, Longhai and Longyan comes from Agriculture and Orchard. Moreover, urban reclamation from Water, especially in the Jiulong River estuary, can explain the systematic transitions from Water to Built-up land in the JRW. Spatially expanding urban growth was identified as one of the leading causes of regional arable land loss in Eastern Coastal China [43,44]. The systematically avoiding transition from Woodland to Built-up was perhaps due to some forest protection policies and laws in China, such as the *Grain for Green* policy implemented since 1999. But the more plausible explanation is that Forest is far away from expanding cities, and Forest is a large dormant category.

The next steps in our research agenda is to check whether the LULC data derived from our methodology are sufficiently accurate to indicate landscape change and the conclusions of Intensity Analysis [45,46].

## 6. Conclusions

This study developed a stratified classification methodology to create a time series of LULC maps in the JRW and analyzed the spatiotemporal dynamics of LULC with Intensity Analysis. The stratified classification produced lower overall error than the unstratified classification. However, the stratified classification produced higher quantity disagreement because the stratified map overestimated Woodland and underestimated Agriculture. The sequence of stratified maps for five time points showed that overall land change in the JRW has been accelerating, which is consistent with accelerating economic development. Woodland is dormant in both gains and losses, while most all other categories are active in both gains and losses, which might be because Woodland accounts most of the JRW, especially in places far from land change. Transitions from Agriculture, Orchard and Water to Built-up are systematically targeting and stationary.

## Figures and Tables

**Table 1.** Characteristics of the satellite images used.

**Date Source**	**Acquisition Date**	**Spatial Resolution(m)**	**No. of Bands**	**Path/Row by Date**	**Source**
Landsat 5TM	7/25/1986, 1/5/1986	25	7	119/43,120/43	CEODE
Landsat 5TM	5/17/1996, 3/5/1996	25	7	119/43,120/43	CEODE
Landsat 7ETM+	1/2/2002, 7/4/2002	30/15	8	119/43,120/43	USGS
Landsat 5TM	2/28/2008, 5/7/2007	25	7	119/43,120/43	CEODE
Landsat 5TM	8/3/2010, 6/6/2009	25	7	119/43,120/43	CEODE

**Table 2.** The LULC classification scheme used in this study.

**No.**	**LULC Types**	**Description**
1	Agriculture	Land used for cultivation, including newly cultivated land, fallow land, swidden land, rotation plough land, land mainly used for planting and beach cultivated more than three years.
2	Orchard	Areas for planting perennial woody plants and perennial herb which were used for collecting their fruit, leaves and rhizome. Cover degree >50%, including Fruit nursery
3	Woodland	Including arbor, bamboo, shrub, Mangrove and pastureland. Residential land use for greening, plants used along railways, highways and rivers are not included.
4	Built-up	Residential area, including the surrounded enterprise area, entertainment area, all kinds of road and airport.
5	Water	Inland water area and water conservancy facilities.
6	Bare land	Unused land, including barren land, wild grass ground, alkaline land, wetland, sand, waste land.
